# Malaria Epidemics and Interventions, Kenya, Burundi, Southern Sudan, and Ethiopia, 1999–2004

**DOI:** 10.3201/eid1210.060540

**Published:** 2006-10

**Authors:** Francesco Checchi, Jonathan Cox, Suna Balkan, Abiy Tamrat, Gerardo Priotto, Kathryn P. Alberti, Dejan Zurovac, Jean-Paul Guthmann

**Affiliations:** * Epicentre, Paris, France;; † London School of Hygiene and Tropical Medicine, London, United Kingdom;; ‡ Médecins Sans Frontières, Paris, France;; § Médecins Sans Frontières, Geneva, Switzerland;; ¶ Kenya Medical Research Institute/Wellcome Trust Research Laboratories, Nairobi, Kenya;; # University of Oxford, Oxford, United Kingdom

**Keywords:** Malaria, Plasmodium falciparum, epidemic, intervention, Kenya, Burundi, Sudan, Ethiopia, perspective

## Abstract

Quantitative data on the onset and evolution of malaria epidemics are scarce. We review case studies from recent African Plasmodium falciparum epidemics (Kisii and Gucha Districts, Kenya, 1999; Kayanza Province, Burundi, 2000–2001; Aweil East, southern Sudan, 2003; Gutten and Damot Gale, Ethiopia, 2003–2004). We highlight possible epidemic risk factors and review delays in epidemic detection and response (up to 20 weeks), essentially due to poor case reporting and analysis or low use of public facilities. Epidemics lasted 15–36 weeks, and patients' age profiles suggested departures from classical notions of epidemic malaria everywhere but Burundi. Although emergency interventions were mounted to expand inpatient and outpatient treatment access, we believe their effects were lessened because of delays, insufficient evaluation of disease burden, lack of evidence on how to increase treatment coverage in emergencies, and use of ineffective drugs.

## Abstract 2

Effectiveness was reduced by delays and other factors.

*Plasmodium falciparum* malaria epidemics were detected in 41 African sites from 1997 through 2002 (*1*). A total of 125 million persons are considered at risk for malaria epidemics, with an estimated yearly death rate of 155,000 to 310,000 (*2*).

Research on malaria epidemics mostly concerns long-range forecasting, early warning, and early detection (improved understanding of the role of temperature, rainfall, and El Niño–Southern Oscillation events [3–6], development of epidemic detection thresholds [7]). Malaria epidemics evolve rapidly and most often occur in remote, underresourced settings without proper surveillance. Data on their evolution may thus go unrecorded, which prevents the development of evidence-based recommendations on effective epidemic control.

Recently, Médecins Sans Frontières (MSF) intervened in several *P. falciparum* malaria epidemics in remote or conflict-affected sub-Saharan African settings. We present case studies from these interventions (Kisii and Gucha Districts, Kenya, 1999; Kayanza Province, Burundi, 2000–2001; Gutten and Damot Gale, Ethiopia, 2003–2004; Aweil East County, southern Sudan, 2003). We also describe the epidemics and possible factors that explain their occurrence, review challenges encountered in their detection and control, and make recommendations for epidemic prevention and control policies. This article reports health facility–based morbidity and mortality data. Findings on deaths in the community will be presented elsewhere (manuscript in review).

## Methods

We reviewed MSF program reports; unpublished assessments (*8**–**12*); and available morbidity, mortality, diagnostic, and treatment data from each of the 5 interventions. We also consulted archives of the United Nations humanitarian data clearinghouse (http://www.reliefweb.int) for general situation reports for each epidemic period and extracted meteorologic indexes to explore the possible contribution of climate to epidemic onset (Appendix).

## Results

### Description of Intervention Sites

Four interventions (Table 1) took place in highland environments, where peaks and valleys create a complex, climate-affected altitude gradient of malaria transmission and age-acquired immunity. Kisii and Gucha Districts are located in the southern highlands of Nyanza Province, Kenya, and experience low year-round transmission, with short, dramatic, and increasingly frequent outbreaks (*16*). Before May 1999, the last recorded epidemic had taken place from January through April 1998. The epidemic we describe also affected 10 nearby districts (*17*).

**Table 1:** Characteristics of intervention sites and potential determinants of epidemics*

Characteristic/ determinant	Kisii/Gucha, Kenya	Kayanza, Burundi	Aweil East, southern Sudan	Gutten, Ethiopia	Damot Gale, Ethiopia
Epidemic period (no. weeks)	May–August 1999 (15)	September 2000–May 2001 (36)	June–November 2003 (22)	July 2003–February 2004 (33)	July 2003–January 2004 (30)
Population	956,000	578,000	307,000	44,000	287,000
Altitude (m)	1,200–2,200	1,400–1,750	430	1,700	1,600–2,100
Malaria vectors	Anopheles funestus (constant), A. gambiae sensu lato (seasonal)	A. arabiensis (95%), A. funestus (5%)	Not available (A. gambiae sensu lato presumed)	A. arabiensis	A. arabiensis
Malaria species (nonepidemic months)	Plasmodium falciparum (>90%)	P. falciparum (>90%)	P. falciparum (>95%)	P. falciparum (≈25%), P. vivax (≈75%)	P. falciparum (≈60%), P. vivax (≈40%)
Temperature anomalies	Above average in 3 preepidemic months	None apparent	Maximum LST strongly below average during epidemic	None apparent	None apparent
Rainfall anomalies	Heavy rainfall in preepidemic rainy season after drought in previous rainy season	Heavy rainfall 5 and 3 months before epidemic, drought 2 years before epidemic but not in preepidemic year	Below average rainfall in 3 preepidemic years, above average in 2 preepidemic months	Below average rainfall in 2 preepidemic and epidemic years but heavy rainfall in preepidemic month	Below average rainfall in 2 preepidemic and epidemic years but heavy rainfall in 3 preepidemic months
Land pattern changes	None reported	Creation of rice paddies and fish ponds	Widespread flooding	Creation of water ponds	None reported
Political instability	None	Armed conflict	Tenuous ceasefire	Inactive insurgency	Inactive insurgency
Population movement	None	Forced relocation	Seminomadic, returnees from north Sudan	Government resettlement schemes	Government resettlement schemes
Global acute malnutrition†	Not available	10%–15%	25%	Not available (probably >5%)	28%
Drug resistance (in vivo failure rates)	CQ 24%–87% (neighboring districts), SP 10% (*13*)	CQ 100%, SP 54.2%, CQ+SP 42.0% (*9*)	CQ 63%, SP 3% (*14*)‡	SP 78.0% (*15*)	SP 68.1% (neighboring zone) (*15*)

*LST, land surface temperature; CQ, chloroquine; SP, sulfadoxine-pyrimethamine. †Among children <5 y of age; malnutrition rates >15% denote a serious situation; values are provided for 2 months before the epidemic. ‡Percentages refer to the frequency of single Pfcrt mutations and triple Dhfr mutations in the P. falciparum genome of outpatients sampled in Aweil East. These mutations are predictive of in vivo CQ and SP failure rates, respectively.

In Burundi's northern Kayanza Province, a 3-year time series up to September 2000 showed constant monthly caseloads of ≈10,000 outpatients/month. In 2000, MSF operated 7 of the province's 22 outpatient facilities. The September 2000–May 2001 epidemic, the largest ever recorded in Burundi, affected 9 of 16 provinces, and 3.5 million cases were reported (*18*).

The Ethiopian highlands experience 2 moderate transmission seasons every year (after rains in March through April and August through September). Epidemics occur in 5- to 8-year cycles; >1 million cases were recorded in 1998 (*1*). The 2003–2004 epidemic affected 15 million persons in 3 federal regions (*19*).

Finally, malaria is considered endemic in low-altitude Aweil East County (Bahr el Ghazal state, southern Sudan), although no data are available. Most cases occur from July through January after spring rains.

### Possible Epidemic Determinants

Findings on possible epidemic determinants are summarized in Table 1. Factors noted at all sites were drought in preepidemic years followed by above-average rainfall in the preepidemic months and elevated drug resistance. Individual sites also experienced temperature abnormalities, land pattern changes, and high malnutrition rates. Further detail on these findings is provided in the Appendix.

### Epidemic Alert and Detection

**Figure 1:**
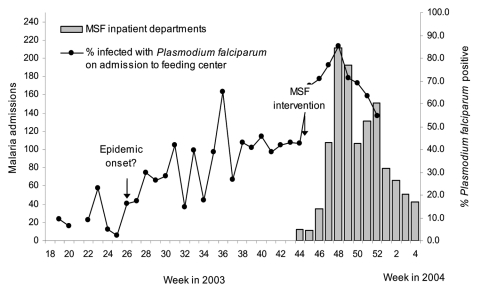
Trends in inpatient malaria caseload and positivity among malnourished children admitted to feeding centers in Damot Gale, Ethiopia, 2003–2004. MSF, Médecins Sans Frontières.

No early warnings were issued. In Kisii, the alert came from the media in epidemic week 5 (when the district hospital was overwhelmed with malaria cases). MSF issued alerts in Kayanza (doubling of fever cases in epidemic week 2, early exhaustion of antimalarial stocks), Aweil East (quadrupled inpatient and outpatient malaria after epidemic week 1), and Damot Gale (increased proportion of *P. falciparum*–positive test results among children admitted to feeding centers, Figure 1). No alert information was found for Gutten.

Formal epidemic declaration was hampered by missing data. Time series for historical comparisons were available in Kisii (12 years) and Kayanza (3 years), where, however, authorities initially suspected a typhus outbreak, until the 80% seroprevalence detected among febrile patients (epidemic week 7) pointed to *P. falciparum*. In Aweil East, comparison with the 2 past years was confounded by 1) a change in diagnostic strategy (from presumptive in 2001 to rapid test–based in 2002 and 2003) and 2) decreased access to treatment in 2003 because of flooding.

In Ethiopia, a malaria-specific surveillance system aimed for early outbreak detection at both the village (positivity >25% detected among slides collected by field workers in the community was considered an outbreak and theoretically led to village-level mass treatment and vector control) and *woreda* or zone (where weekly reports from health facilities were compiled) levels. A malaria epidemic was declared in August 2003 (6–10 weeks after probable onset) by East Wollega Zone, including Gutten. Data from this surveillance system were difficult to interpret. Only some of the reports reached the zone bureau, with a delay of 4 to 6 weeks. In Damot Gale, despite incomplete reporting, a massive increase in caseload was evident at the province level (6,500 from July through October 2003 vs. 1,233 from July through October 2002); however, by early July (epidemic onset), only reports up to April were available. Furthermore, clinics aggregated mass fever treatment and outpatient data, causing artificial incidence spikes when the former took place. Conversely, drug shortages in August gave a false impression of declining incidence. MSF had only been present in Damot Gale since April 2003 and only operated feeding centers.

### Operational Response

Interventions occurred 3–20 weeks late (Table 2). In Kisii and Gucha, numerous agencies, including Merlin, African Medical and Relief Foundation (AMREF), Cooperative for Assistance and Research Everywhere, Inc. (CARE), World Vision, the Kenyan Army, and medical staff of Kenyatta National Hospital supported health structures. Elsewhere, MSF was the sole external agency. Everywhere, facilities were initially overwhelmed. In Kisii Hospital, bed occupancy reached 300% in the first 3 weeks. Hospitalization capacity was 0.45 beds per 10,000 people in Gutten and 0.53 beds per 10,000 in Damot Gale, where, in June and July 2003 (epidemic onset), the rate of outpatient consultations per person per year was 0.03–0.09 (0.5–1.0 is expected in such settings if access is good) (*20*). Although waived once epidemics were officially recognized, user fees were initially charged in Kenya, Burundi, and Ethiopia. In Aweil East, non-MSF clinics had run out of chloroquine 3 weeks into the outbreak.

**Table 2:** Details of operational response to malaria epidemics by intervention site*

Factor		Kisii/Gucha, Kenya	Kayanza, Burundi	Aweil East, southern Sudan	Gutten, Ethiopia	Damot Gale, Ethiopia
Delay of intervention (wks)		7	7	3	>12, probably 19	20
Inpatient care						
	Expansion in bed capacity	From 310 to 510 beds	From 65 to 125 beds	From ≈80 to ≈120 beds	From 2 to ≈100 beds	From 12 to >100 beds
	Treatment	IM/IV quinine, IM artemether	IM/IV quinine	IM artemether	IV quinine	IV/IR quinine
	Diagnosis	Presumptive	Blood slide	RDT	RDT	RDT
Fixed outpatient care						
	Increase in capacity	2 additional OPDs	Increased capacity in 5 OPDs, 2 additional OPDs	Conversion of nutritional centers, 2 additional OPDs	1 additional OPD	Supervision and drug supply to 5 OPDs
	Treatment	SP	CQ+SP	AS+SP	Quinine (IR if vomited)	SP, quinine
	Diagnosis	Presumptive	Presumptive	RDT	RDT	RDT
Mobile clinics						
	Number	3	6	14	5	Not available
	Catchment population	302,000	Not available	144,000	44,000	73,000
	Sites visited	45	10	43	5	14
	Days per site per week (wks of operation)	0.2–0.3 (7)	1.2 (22)	1–2 (15)	2 (13)	0.2–0.5 (4)
	Treatment	SP, AS+SP (73.4% of cases)	CQ+SP	AS+SP, artemether for severe cases	Quinine	Quinine
	Diagnosis	Presumptive	Presumptive	Presumptive	RDT	RDT

*IM, intramuscular; IV, intravenous; IR, intrarectal; RDT, rapid diagnostic test; OPD, outpatient department; SP, sulfadoxine-pyrimethamine; CQ, chloroquine; AS, artesunate.

All interventions included inpatient components with blood transfusion. Conversion of existing MSF nutritional structures enabled expansion of care in Aweil East and Damot Gale. To reach isolated communities, mobile clinics, consisting of teams of nurses or nursing assistants working with simple treatment algorithms, were established at each site. However, this intervention occurred late (10 weeks late in Kisii and Gucha, 7 in Kayanza, 8 in Aweil East, 13 in Gutten, and 27 in Damot Gale) and, apart from in Kayanza, after the epidemic peak (Table 2). Choice of location depended on results of a cross-sectional prevalence survey (Kisii and Gucha), distance from the nearest health center or proximity to swampy areas (Kayanza), known gathering point and greatest distance to the outpatient department (Aweil East), known gathering point near existing health posts (Gutten), and village morbidity/mortality surveillance results (Damot Gale). Diagnosis was presumptive everywhere except Ethiopia, where the *P. falciparum*–specific rapid diagnostic test (RDT) Paracheck (Orchid Biomedical Systems, Verna, Goa, India) was used systematically (Table 2). In Aweil East, mobile teams traveled on bicycle and canoe, spending 3–4 days in each location; because transporting patients with severe cases was impossible, more experienced teams carried injectable artemether and anticonvulsant drugs and treated 110 patients on a semi-inpatient basis (no outcome was recorded for these patients). At other sites, mobile clinics remained on site for 1 day and provided an ambulance service. Mobile teams were present in each targeted village for no more than 1–2 days a week on average and as little as once a month in Kenya and Damot Gale (Table 2). Mobile clinics treated 46,541 (9.3%) of 501,214 reported cases in Kayanza, 34,749 (68.3%) of 50,863 in Aweil East, 7,258 (19.4%) of 37,457 in Gutten, and 467 (2.8%) of 16,621 in Damot Gale (Table 3). In Damot Gale, active severe case finding was organized (no data available).

**Table 3:** Epidemiologic profile of malaria at fixed inpatient, fixed outpatient, and mobile health facilities operated by Médecins Sans Frontières in 5 intervention sites

Characteristic			Kisii/Gucha, Kenya	Kayanza, Burundi	Aweil East, southern Sudan	Gutten, Ethiopia	Damot Gale, Ethiopia
Uncomplicated cases							
	Fixed outpatient centers						
		All ages	13,127*	272,459	15,239	15,928†	–
		Age <5 y (%)	2,426 (18.5)	Not available	7,257 (47.6)	4,758‡ (29.9)	–
	Mobile clinics						
		All ages	29,769	46,541	34,749	7,258	467
		Age <5 y (%)	5,376 (18.1)	Not available	17,338 (49.9)	1,405 (19.4)	145 (31.0)
Complicated cases							
	All ages		9,773§	3,953¶	875#	330**	1,291
	Age <5 y (%)		5078 (52.0)	761 (19.3)	683 (78.1)	175 (53.0)	595 (46.1)
	No. deaths (CFR [%])		397 (4.1)	108 (2.7)	50 (5.7)	34 (10.3)	62 (4.8)
	No. deaths <5 y (CFR [%])		164 (3.2)	31 (4.1)	39 (5.7)	15 (8.6)	38 (6.4)
Minimal attack rate (%)††			22.2 (complicated, <5 only; 12/15 weeks)	86.5 (36/36 weeks)	41.2 (<5 only; 22/22 weeks)	53.4 (15/33 weeks)	Not available
P. falciparum prevalence at epidemic peak (%)			38–49 (community survey)	80 (random sample in OPD queue)	52–64 (random sample in OPD‡‡ queue)	Not available	60 (random sample by community workers)

*Includes data from 3 government clinics (Masimba, Kenyenya, and Etago) for which age breakdown was available. †Includes 2,061 patients treated with intrarectal quinine in inpatient department. ‡Includes 1,773 patients <5 years of age treated with intrarectal quinine in inpatient department. §Includes data from Kisii, Keumbu, and Ogembo hospitals, supported by Médecins Sans Frontières and other agencies but operated by the government. ¶Excludes patients treated in the Kayanza government hospital (data not available). #Excludes 110 severe cases treated by mobile clinics (no age breakdown or outcome available). **Includes only hospitalized patients who met a strict definition of severe malaria, which probably explains the considerably higher case-fatality ratio (CFR) noted in Gutten. ††Ratio of weeks refers to the number of epidemic weeks from which the attack rate was calculated divided by the total number of epidemic weeks. ‡‡OPD, outpatient department.

Artemisinin-based combination therapy (ACT) was deployed in Aweil East and in mobile clinics in Kenya (Table 2). Its use was not officially authorized in Burundi and in Ethiopia, where empiric evidence of poor sulfadoxine-pyrimethamine efficacy, later confirmed by in vivo studies (*15*), led clinicians to use quinine as first-line treatment. To ensure adherence to the 7-day regimen, high-risk patients were treated intrarectally under observation (Table 3).

### Surveillance and Epidemic Evolution

In Burundi, Sudan, and Ethiopia, surveillance data were analyzed weekly. In Kayanza, RDT testing was carried out every 2–3 weeks among outpatients to monitor epidemic trends. In Aweil East and Gutten, an automated surveillance spreadsheet generated key indicators and graphs (caseload, proportionate morbidity and mortality, case-fatality, RDT confirmation of diagnosis).

**Figure 2:**
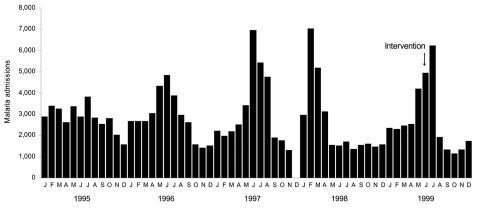
Trends in outpatient malaria caseload in Kisii Hospital outpatient department, Kenya, 1995–1999. Data for December 1997 are missing because of a nursing staff strike.

The Kisii and Gucha epidemic followed a historical pattern of short dramatic peaks (Figure 2). Kisii Hospital records showed that, during the first 12 epidemic weeks, 2,669 (22.2%) of children <5 years of age in Kisii municipality (≈12,000) were hospitalized for malaria (Table 3).

**Figure 3:**
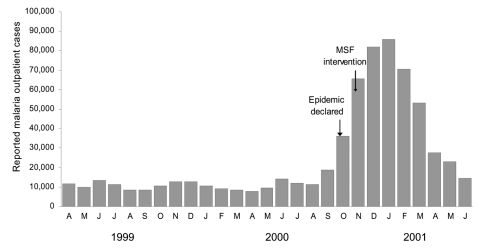
Trends in outpatient malaria caseload in Kayanza Province, Burundi, 1999–2001. MSF, Médecins Sans Frontières.

The Kayanza epidemic lasted 36 weeks and roughly followed a normal distribution (Figure 3). A total of 501,214 cases were reported, for a minimum attack rate of 86.5%.

**Figure 4:**
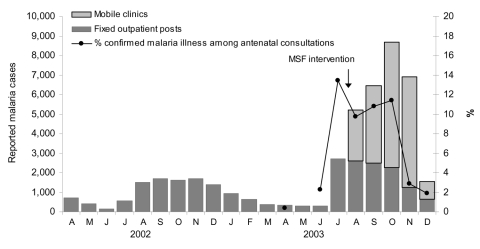
Trends in outpatient caseload and proportionate malaria among pregnant women attending antenatal consultations in Aweil East, southern Sudan, 2002–2003. MSF, Médecins Sans Frontières.

In Aweil East, a peak was reached by epidemic week 2, and a steady decline followed, which reflected percentage of confirmed malaria cases among women who came to the clinic for antenatal visits (Figure 4). Children <5 years of age (assumed to be 20% of the population) experienced attack rates of >41.2% (all malaria) and 1.1% (complicated).

**Figure 5:**
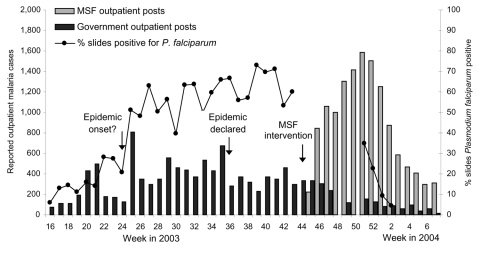
Trends in outpatient malaria caseload and slide positivity in Gutten, Ethiopia, 2003–2004. MSF, Médecins Sans Frontières.

In Ethiopia, the epidemic's evolution can partly be reconstructed by plotting available microscopy results from the Gutten government clinic, which yields a normal distribution (Figure 5), and percentage *P. falciparum* positivity among malnourished children admitted to feeding centers in Damot Gale (Figure 1). Results showed a steady rise from June, a plateau in August and September, and a new peak in late November after heavy rains.

### Profile of Patients

Among uncomplicated cases, the proportion of patients <5 years of age exceeded the expected levels of 15% to 20% in southern Sudan and Ethiopia but not Kenya, where only presumptive diagnosis was used (Table 3). Patients <5 years made up half of all complicated cases in Kisii, Gucha, Gutten, and Damot Gale and almost 80% in Aweil East. Case-fatality rates were comparable across ages and sites except Gutten (footnote, Table 3). Convenience samples of outpatients or household surveys suggested high *P. falciparum* prevalences at or near caseload peaks (Table 3). Where MSF was involved in both outpatient and inpatient care (Kayanza, Aweil East, and Gutten), comparable proportions of patients were hospitalized (1.2%, 1.9%, and 1.4% respectively).

## Discussion

In sub-Saharan Africa, malaria epidemics arise suddenly in mostly remote, disadvantaged settings without effective alert systems. Our case studies show that large-scale interventions can be organized in such epidemics, and that these interventions can considerably increase diagnostic and treatment output. Both preparedness and control, however, were seriously deficient. Epidemic detection was late everywhere, and additional delays occurred before external intervention to support overwhelmed local health structures.

Experiences in Kisii, Gucha, Kayanza, Gutten, and Damot Gale probably reflect conditions in neighboring regions affected by the corresponding epidemics, although scarcity of published records makes comparisons difficult. This analysis relies on programmatic data, the limitations of which are apparent.

## Epidemic Risk and Vulnerability Factors

### Climate

Our analysis did not include controls (i.e., sites where no epidemics occurred). Nevertheless, remotely sensed climate data suggest rainfall abnormalities during key preepidemic periods: relative drought in the 2 or 3 preepidemic years (with the exception of Kayanza) and above-average rainfall 1–2 months before epidemic onset. No consistent temperature pattern emerged.

The full role of such abnormalities as epidemic determinants is unclear. Furthermore, although remotely sensed environmental variables provide relatively robust and accurate estimates (*21*) and are becoming more publicly accessible (*22*), they remain only proxies for ground-based measurements. Nonetheless, we believe that our findings implicate climate abnormalities to a varying extent in all 5 epidemics and support strengthened monitoring of climate variables for early warning.

### Other Factors

Land cover changes in Aweil East (flooding) and in Kayanza (rice paddy creation) probably favored vector breeding. Malnutrition, displacement, and drug resistance may not in themselves cause epidemics, but in our settings these factors probably exacerbated the epidemics' magnitude, duration, and case-fatality ratios. The effects of past drought and malnutrition are difficult to extricate: they are related causally, and either could result in impaired immunity (respectively, through reduced exposure to infection and nutrient deficiencies).

To our knowledge, no entomologic data were collected during any of these epidemics, which limits the strength of our findings; the role of changes in vector species or breeding habitats could have had a major role, but these factors can only be imputed from observed land pattern or climate alterations. Future studies on malaria epidemics should include detailed entomologic profiling, even during the epidemics.

In short, we believe that, given available evidence, to predicate epidemic prevention activities solely on the basis of individual risk factors (meteorologic or other) would be imprudent. Instead, appropriate decision support systems should be built that integrate all relevant data (e.g., environmental variables, food security and nutritional status, drug efficacy, health coverage, vector characteristics, population at risk) into a risk profile for each epidemic-prone population, to be updated regularly; in such a scenario, warning flags (*23*) resulting from detected environmental anomalies or other risk factors would result in enhanced surveillance activity and increased emergency preparedness (e.g., stockpiling drugs, ensuring insecticides and spray teams are in place), rather than leading directly to control activities.

## Difficulties in Detection

Even without early warning, detecting epidemics within 2 weeks of onset should be possible (*24*), provided that weekly reporting and analysis are complete and timely and that caseload data reflect community incidence trends. In most case studies described here, epidemics were detected after substantial delay and by agencies other than local authorities with unconventional methods, such as RDT monitoring among malnourished children. An exception was Aweil East, where weekly reporting and analysis took place. Several formal definitions of a malaria epidemic have been proposed. Most, like the C-Sum or Cullen methods (*24*), rely on comparison with past caseloads. These methods cannot be effective unless surveillance is greatly improved. Experience from the Highland Malaria Project (HIMAL) (*7*) shows that meeting the requirements of epidemic early detection requires supplementing existing routine surveillance systems with networks of representative sentinel health facilities, new data collection forms, procedures for rapid exchange of data between different levels of the health system, and tools for the collation, analysis, and interpretation of incoming data. However, the apparent success of HIMAL's pilot early detection systems in selected districts of Uganda and Kenya suggests that these efforts are viable, given requisite resources and motivation (J. Cox and T. Abeku, pers. comm.).

Free treatment and steady drug supplies probably favored early detection in Aweil East. Conversely, in Ethiopia, facility use was too low to reflect the magnitude of the emergency, and irregular drug distributions confounded epidemiologic monitoring. User fee systems may have long-term benefits, but cost barriers hamper treatment access (*25*). In impoverished populations at risk for malaria epidemics, a free care policy may encourage early treatment seeking and thus facilitate early epidemic detection and monitoring, in addition to minimizing deaths. Conversely, user fee systems may result in "silent" public health disasters.

## Intervention Strategies

### Expansion of Access

By the time interventions were implemented, their potential effects were reduced. Mobile clinics were deployed to expand health access and detect severe cases. Implementation of clinics understandably varied according to local conditions, but apart from in Aweil East, probably had limited impact. Mobile clinic programs should be designed on the basis of clearly identified catchment areas and set frequencies with which communities should be visited. Although various criteria were used in our case studies, we believe that actual access to healthcare should be a key indicator for selecting target populations. Rapid methods to assess antimalarial treatment coverage thus need to be developed. How frequently communities are visited determines both the improvement in treatment coverage and the probability of preventing progression to severe disease through prompt treatment, which is likely to increase exponentially with frequency of mobile team visits; we hypothesize that frequent visits to selected sites may be more efficient than infrequent visits to a wider area. Impact monitoring should be included in future mobile clinic interventions to adjust their strategy as the situation evolves, and they should be evaluated after the fact. More generally, alternative modes of rapidly decentralizing care, such as fixed temporary health posts or training of resident community health workers (possibly equipped with artesunate suppositories to treat severely ill patients), merit further exploration. Where no clear indications exist that local health structures can cope with a large malaria epidemic, mobile clinics or other temporary treatment programs should be implemented immediately.

### Reduction of Case-fatality Ratio

Case-fatality ratio among patients with complicated cases was lower than current best estimates of 10% (*2*) and 13% (*26*); however, whether all cases were severe depends on the case definition used. Treatment of uncomplicated cases relied on failing drugs everywhere but Sudan, and sulfadoxine-pyrimethamine monotherapy was probably counterproductive because the drug stimulates gametocytogenesis (*27*) and thus transmission. Ineffective drug use in Burundi probably limited the effect on mortality; in Ethiopia, quinine first-line administration proved challenging because of vomiting and required impractical patient monitoring.

### Effect on Public Health

Kayanza excluded, the increased proportion of children <5 years of age among inpatients, as previously observed in Kenya (*28*), suggests that children were more susceptible to symptomatic disease, which challenges classical notions of unstable, epidemic malaria. In Aweil East, the predominance of children is consistent with stable, mesoendemic to hyperendemic transmission, and this situation is probably better characterized as a severe seasonal outbreak.

Clinic-based attack rates approach 100% for all age groups when extrapolated to the entire epidemic period (Kayanza and Gutten) and are even more alarming among children <5 years of age in Kisii. Even after overdiagnosis from presumptive treatment is accounted for, these rates are likely to be gross underestimates. The vast gap in treatment coverage was evident in Aweil East, where large-scale deployment of mobile clinics greatly increased output, and in Ethiopia, where despite capturing only the declining phase of the epidemic, uninterrupted provision of free care with effective drugs resulted in far higher outpatient and inpatient department attendance. The true community incidence in these epidemics is probably much greater than represented by regular reporting systems and higher than current estimates of 0.5 episodes of malaria per person per epidemic (*29*). Only population-based studies can yield realistic estimates of this incidence.

## Conclusion

Malaria epidemics create daunting medical emergencies. In addition to ongoing research on alert systems, much greater donor investment is necessary to prevent and control them. All 4 countries in this study are moving to ACT combinations for outpatient treatment, a major improvement that is still insufficient unless 1) simple but valid surveillance data are transmitted and analyzed on a weekly basis, maximizing the chance of early epidemic detection, and 2) treatment coverage of uncomplicated and complicated cases truly reflects community needs. Further research is needed on methods to rapidly estimate needs (incidence) and coverage and on strategies to efficiently expand treatment access. Arguably, focusing resources only on how to predict and respond to epidemics might lead policymakers to overlook basic problems with access to effective treatment and tools for prevention that are common to both epidemic and stable malaria settings and that probably merit similar solutions. Donors and policymakers should thus aim for a balanced approach: improved capacity for epidemic prediction and response is needed, but long-term improvements in access to proper care and vector control by all members of the community, even before epidemics strike, must not be neglected, as they could be the most relevant determinants of decreased epidemic severity.

Because malaria epidemics are difficult to predict and multifactorial, setting up controlled studies to formally demonstrate the benefit of any single intervention will be difficult. Properly documenting the cost, feasibility, and output of these interventions and measuring the true extent of malaria epidemics are nevertheless crucial to inform the choice of future prevention and control strategies and must be included in the research agenda.

## Appendix

### Additional Information on Climate and Other Possible Epidemic Determinants

We characterized climatic and ecologic conditions during each epidemic by using meteorologic indexes obtained by remote sensing (station data were incomplete or unavailable). Dekadal (10-day) rainfall estimates for July 1981 through December 2005 and normalized difference vegetation indexes for July 1995 through December 2005 were obtained at 8-km spatial resolution from the Africa Data Dissemination Service (http://igskmncnwb015.cr.usgs.gov/adds/index.php). Data for daytime and nighttime land-surface temperature (LST) were obtained through the Epidemio project (http://www.epidemio.info/index.php?section=homepage) for January 1995 through September 2005 at 2.5-minute spatial resolution. For each site, administrative boundaries were overlaid with remotely sensed datasets in a geographic information system with Arc/Info workstation software (version 8.1, ESRI, Redlands, CA, USA). We extracted summary statistics for each parameter and dekad (10-day period) and compared these with long-term averages for the same season. Our aim was to identify obvious climate anomalies either during or before the epidemics; therefore, we limited ourselves to descriptive analysis rather than establishing causality between climate and epidemic onset.

### Climate

In Kisii, estimated rainfall from September 1998 through January 1999 was 59% below the expected (536 mm), according to the 10-year rainfall estimate dataset. The long rains of March and April 1999 (1 month before epidemic onset) were unusually heavy (704 mm compared with a long-term average of 401 mm). Land-surface temperature for 1998 and 1999 generally fell within the normal range from 1995 through 2005, except during the preepidemic period, November 1998 through February 1999, when average maximum land-surface temperature (33.1°C) was 14% higher than the corresponding long-term average.

In Kayanza, unusually heavy rains occurred between the second dekad of February and the first dekad of April 2000 (460 mm vs. a 1995–2005 average of 304 mm), and an additional peak occurred during the last dekad of May (137 mm vs. 22 mm), 3 months before epidemic onset. Data from neighboring Ngozi Province suggested a rise in temperature (*1*), but no land-surface temperature anomalies are evident for this period.

In East Wollega Region, including Gutten Locality, 2002 and 2003 show similar drought patterns from the last dekad of April to the end of May (61 mm and 33 mm, respectively, compared with an average of 192 mm from 1995 through 2005), followed by above-average rainfall throughout June (preepidemic month), especially in 2003. Annual rainfall estimate averages for 2001 through 2003 (962 mm) seem markedly drier than preceding years (1996–2000, 1,431 mm). Drought conditions in the 2003 preepidemic months are also captured by mean normalized difference vegetation index data (0.38 from the last dekad of April to end of June 2003 vs. 0.5 average for the same period [1995–2005]). No land-surface temperature anomalies were apparent.

In Damot Gale, annual rainfall estimate averages from 2001 through 2003 (940 mm) were lower than for 1996 to 2000 (average 1,204 mm). In the preepidemic months of April, May, and June, rainfall estimate was 268 mm in 2002 (preepidemic year), compared with 376 mm in 2003 (epidemic year) and the 1996–2004 mean of 416 mm. land-surface temperature for this period seemed within normal range.

In Aweil East, 2000–2002 was relatively dry, particularly in 2002 when annual rainfall estimate was 40% below the long-term average (963 mm). Rainfall totals for 2003, however, were ≈20% higher, with a particularly rainy season leading up to May (preepidemic month). Land-surface temperature data suggest that temperatures in the second half of 2002 and first part of 2003 were within normal range, but during the epidemic period itself (June–November), mean maximum land-surface temperature (34.1°C) was 3.5°C below the period average.

### Other

All sites but Kenya were affected by conflict and displacement. Burundi's and southern Sudan's conflicts had lasted 7 and 20 years, respectively; Aweil East's population had been repeatedly displaced by militia incursions. Both Gutten and Damot Gale had received immigrants resettled from drought-stricken, nonmalarious areas.

Parasite resistance to first-line treatments was high in Kayanza (chloroquine [CQ]), Sudan (CQ), and Ethiopia (sulfadoxine-pyrimethamine [SP] at the peripheral level and the combination CQ+SP in better-equipped centers). SP was prescribed as first-line in Kenya and Ethiopia (primaquine coadministration was mandated by Ethiopian guidelines, but this drug was not supplied to Gutten and Damot Gale during the epidemic).

### Appendix Reference

1. Bonora S, De Rosa FG, Boffito M, Di Perri G, Rossati A. Rising temperature and the malaria epidemic in Burundi. Trends Parasitol. 2001;17:572–3. 10.1016/S1471-4922(01)02095-511756033
